# Anifrolumab for rituximab-induced SCLE and cryoglobulinemic vasculitis in primary Sjögren’s disease: a case report

**DOI:** 10.3389/fphar.2026.1856073

**Published:** 2026-08-17

**Authors:** I. Magi, M. Bellinvia, V. Caputo, F. Rongioletti, C. Bochicchio, M. R. Pellico, R. Caporali, N. Del Papa

**Affiliations:** 1 Department of Clinical Sciences and Community Health, University of Milan, Milan, Italy; 2 Centro Medico Bellinvia, Milan, Italy; 3 Department of Surgical Pathology, ASST Grande Ospedale Metropolitano, Milan, Italy; 4 Dermatology Clinic, IRCCS San Raffaele Hospital, Milan, Italy; 5 Department of Rheumatology and Medical Sciences, ASST Gaetano Pini-CTO Institute, Milan, Italy

**Keywords:** case report, drug-induced lupus, rituximab, Sjögren’s disease, subacute cutaneous lupus erythematosus

## Abstract

Rituximab, a chimeric anti-CD20 monoclonal antibody, is widely used to treat autoimmune diseases and hematologic malignancies through B-cell depletion, yet paradoxical autoimmune phenomena, including drug-induced lupus erythematosus, have been increasingly recognized. While paradoxical autoimmune phenomena following rituximab therapy have been reported, drug-induced subacute cutaneous lupus erythematosus (DI-SCLE) represents an exceedingly rare complication. This case reports rituximab-induced SCLE in a patient with Sjögren’s disease and demonstrates the dual therapeutic efficacy of anifrolumab in managing both drug-induced cutaneous lupus and interferon-driven systemic manifestations. A 69-year-old woman with established primary Sjögren’s disease and cryoglobulinemic vasculitis developed biopsy-proven subacute cutaneous lupus erythematosus with clinical onset approximately 4 months following rituximab administration, with histopathological confirmation at Month 9. The patient presented with characteristic erythematous lesions on the trunk and back, which subsequently recurred with extension to the face and décolleté; histopathological examination revealed interface dermatitis and a positive lupus band test on direct immunofluorescence. Treatment with anifrolumab (300 mg intravenously every 4 weeks) resulted in complete resolution of rituximab-induced SCLE. Subsequent addition of low-dose mycophenolate mofetil (1,000 mg daily) achieved complete remission of cryoglobulinemic vasculitis, with sustained disease control maintained on this anifrolumab-based regimen. Clinicians should maintain vigilance for paradoxical cutaneous reactions following B-cell-depleting therapies, particularly in patients with underlying Sjögren’s disease and anti-Ro/SSA positivity.

## Introduction

Rituximab, a chimeric monoclonal antibody targeting CD20-positive B lymphocytes, has revolutionized the treatment of various autoimmune and hematologic disorders (Edwards and Cambridge, 2006). Its mechanism of action through selective B-cell depletion has proven efficacious in conditions such as rheumatoid arthritis, systemic vasculitis, and lymphoproliferative malignancies (Stone et al., 2010). In the context of Sjögren’s disease, rituximab demonstrates particular utility in managing extraglandular manifestations, including cryoglobulinemic vasculitis (Devauchelle-Pensec et al., 2014). Despite its established therapeutic role, rituximab has been paradoxically associated with the development of new autoimmune phenomena (Kowal-Bielecka et al., 2013). The occurrence of drug-induced lupus erythematosus (DILE) following rituximab therapy represents a rare but clinically significant complication (Lowe et al., 2011). Among DILE variants, subacute cutaneous lupus erythematosus (SCLE) is characterized by distinctive photosensitive skin lesions and specific histopathological features, including interface dermatitis and positive lupus band test on direct immunofluorescence (Lowe et al., 2011). Importantly, SCLE pathogenesis is strongly associated with activation of the type I interferon pathway, with interferon-signature genes consistently upregulated in cutaneous lesions (Somers et al., 2012; Braunstein et al., 2012).

Primary Sjögren’s disease, particularly in its systemic manifestations, is similarly characterized by prominent type I interferon pathway activation (Brkic et al., 2013; Maria et al., 2015). Elevated interferon-regulated gene expression correlates with disease activity and extraglandular involvement, including cryoglobulinemic vasculitis (Maria et al., 2015). This shared interferon-driven pathophysiology between Sjögren’s disease and SCLE suggests that patients with pre-existing Sjögren’s disease may be particularly susceptible to developing interferon-mediated autoimmune complications following immunomodulatory therapies (Maria et al., 2015; Erazo-Martínez et al., 2023).

Anifrolumab, a fully human monoclonal antibody targeting the type I interferon receptor subunit 1 (IFNAR1), represents a targeted therapeutic approach for interferon-driven autoimmune diseases (Furie et al., 2017). By blocking type I interferon signaling, anifrolumab has demonstrated efficacy in systemic lupus erythematosus, including significant improvement in cutaneous manifestations (Furie et al., 2019; Morand et al., 2020). Limited evidence suggests potential benefit in other interferon-mediated conditions, though data in Sjögren’s disease remain sparse (Chasset et al., 2021).

The pathophysiological mechanisms underlying rituximab-induced autoimmunity remain incompletely elucidated. Proposed hypotheses include dysregulation of immune homeostasis following B-cell depletion, altered regulatory T-cell function, and the unmasking of latent autoimmune predisposition (Carter et al., 2013). The temporal relationship between rituximab administration and SCLE onset, typically ranging from weeks to months, supports the notion of a complex immunological cascade rather than a direct drug-mediated effect (Devauchelle-Pensec et al., 2014).

This case report describes a patient with primary Sjögren’s disease who developed biopsy-confirmed SCLE with clinical onset approximately 4 months after rituximab therapy for cryoglobulinemic vasculitis, with histopathological confirmation at Month 9. The case is notable for demonstrating the therapeutic efficacy of anifrolumab in resolution of rituximab-induced SCLE and, as part of an anifrolumab-based regimen, in achieving complete remission of Sjögren’s-associated vasculitis refractory to B-cell depletion. This report adds to the limited literature on rituximab-associated cutaneous lupus and provides a documented example of anifrolumab use in this rare complication (Lowe et al., 2011).

## Case presentation

**FIGURE 1:**
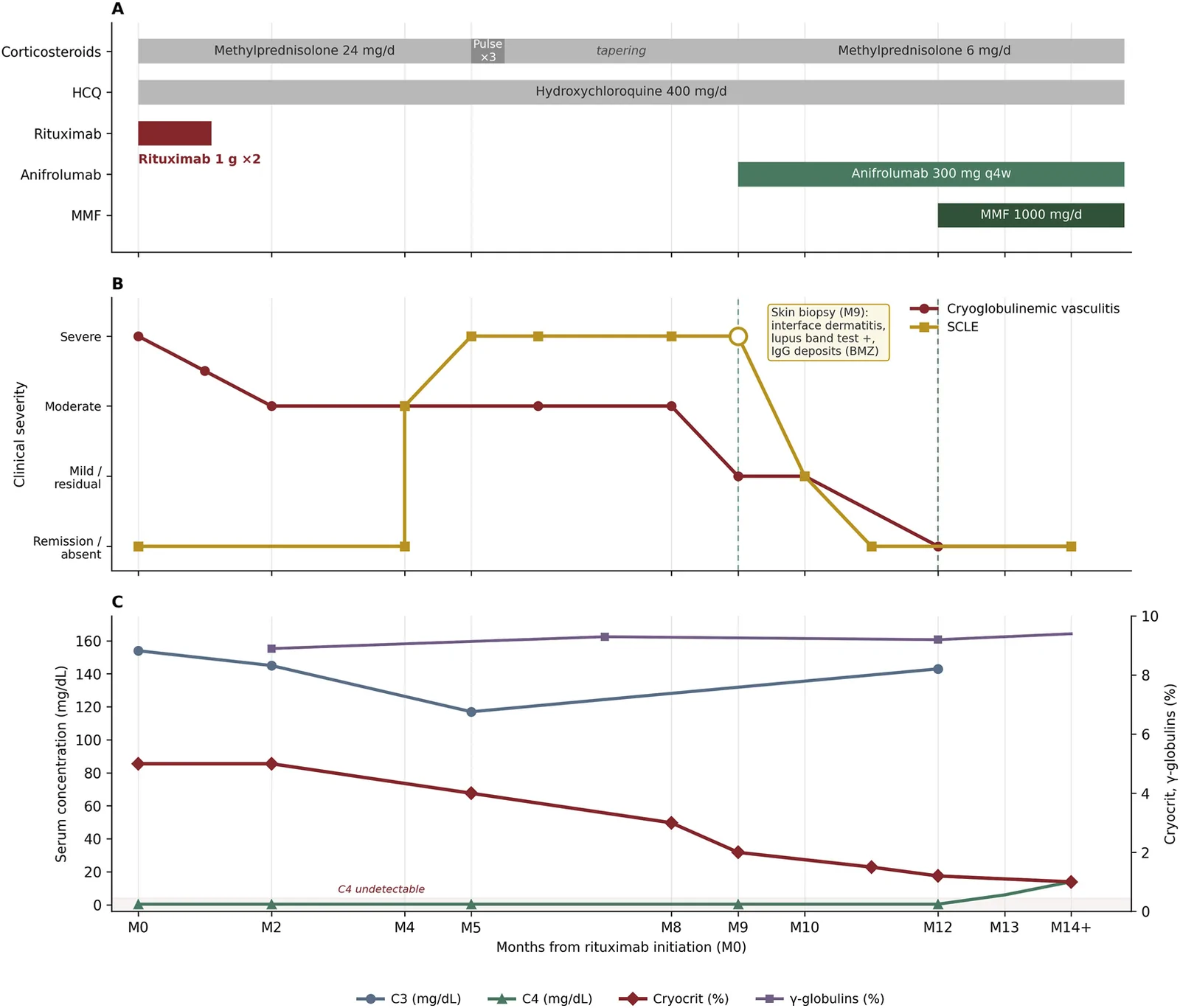
Clinical course and treatment timeline (months from the first rituximab infusion, M0). **(A)** Treatments. **(B)** Disease manifestations graded by clinical severity; the M9 skin biopsy confirming SCLE (interface dermatitis, positive lupus band test with basement-membrane IgG deposits) is indicated. **(C)** Serial laboratory parameters: the progressive fall in cryocrit paralleled improvement of the cryoglobulinemic vasculitis, with cutaneous and vasculitic remission achieved on the anifrolumab-based regimen. HCQ, hydroxychloroquine; MMF, mycophenolate mofetil; SCLE, subacute cutaneous lupus erythematosus.

A 69-year-old Caucasian woman with established primary Sjögren’s disease presented with progressive erythematous cutaneous lesions developing with clinical onset approximately 4 months after rituximab therapy for cryoglobulinemic vasculitis (histopathological confirmation obtained at Month 9, as detailed below). The diagnosis of Sjögren’s disease had been established 3 years and 3 months before the initiation of rituximab and fulfilled the 2016 ACR/EULAR classification criteria, based on sicca symptoms (xerophthalmia and xerostomia), recurrent parotid swelling, arthralgias, positive minor salivary gland biopsy (lymphocytic sialadenitis with focus score consistent with Sjögren’s disease), negative salivary flow rate assessment, and positive Schirmer’s test, in association with serological positivity for anti-SSA/Ro, anti-SSB/La, and weakly positive rheumatoid factor. She had associated type II cryoglobulinemia and no prior history of relevant ultraviolet exposure or photosensitivity. Previous azathioprine had been discontinued due to hepatotoxicity, and the disease had been well controlled with hydroxychloroquine 400 mg/day until vasculitic manifestations occurred. Clinical timeline and disease course are summarized in Figure 1.

At Month 0, she developed palpable purpura on the lower extremities consistent with cryoglobulinemic vasculitis. Laboratory tests showed undetectable C4, normal C3 (154 mg/dL), and cryoglobulins with a cryocrit of 5%; screening for hepatitis B, hepatitis C, and tuberculosis (QuantiFERON) was negative. Methylprednisolone 24 mg/day produced only modest improvement, leading to initiation of rituximab 1,000 mg intravenously on Days 1 and 15 according to standard autoimmune protocols, which was well tolerated. At Month 2, purpura had partially improved, but vasculitic activity persisted, with elevated erythrocyte sedimentation rate (43 mm/h), negative C-reactive protein, persistently undetectable C4, C3 of 145 mg/dL, and gamma globulins of 8.9%.

**FIGURE 2:**
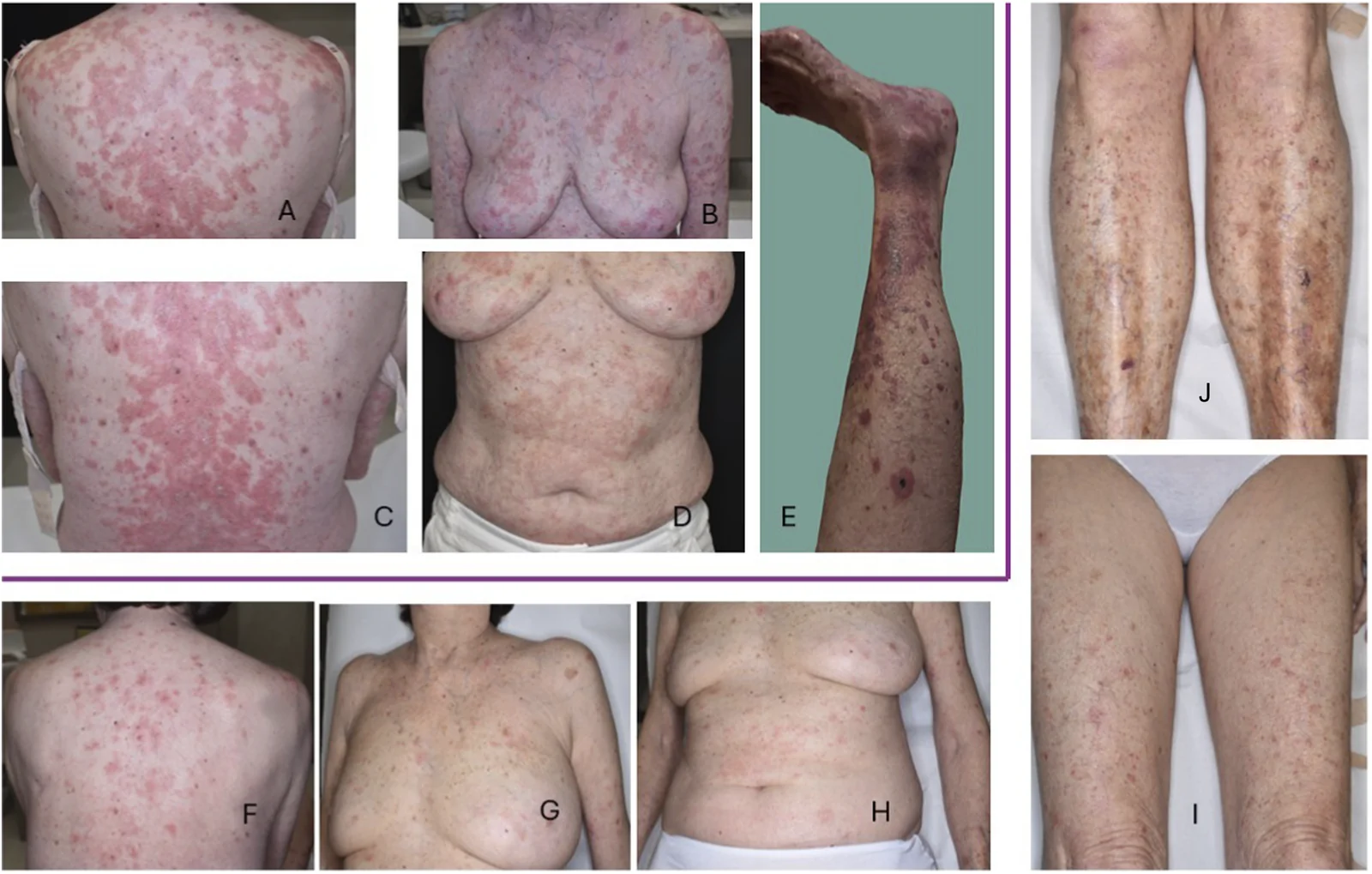
Clinical response to anifrolumab therapy in rituximab-induced subacute cutaneous lupus erythematosus. **(A–E)** Clinical presentation prior to anifrolumab initiation, showing extensive erythematous annular plaques and patches on the upper back **(A)**, anterior chest and décolleté **(B)**, posterior trunk **(C)**, anterior abdomen **(D)**, and associated cryoglobulinemic vasculitis with purpuric lesions on lower extremity **(E)**. **(F–J)** Clinical appearance at 4 weeks following anifrolumab 300 mg intravenous infusion with hydroxychloroquine 400 mg daily, demonstrating marked improvement with near-complete resolution of erythematous plaques on the posterior back **(F,G)**, anterior chest and trunk **(H)**, anterior thighs **(I)**, and significant improvement of vasculitic purpura on lower extremities **(J)**. Note the photodistributed pattern of cutaneous lesions predominantly affecting sun-exposed areas and the dramatic therapeutic response to type I interferon blockade.

At Month 4, she developed new erythematous papules and plaques on the trunk and back, clinically distinct from prior vasculitic lesions, raising clinical suspicion for drug-induced SCLE. Two weeks after onset, dermatologic examination confirmed photodistributed erythematous lesions on the trunk and dorsal regions (Figures 2A–D). By Month 5, the rash had progressed, becoming more confluent and extensive over the trunk, chest, and back (Figures 2E–I), prompting a diagnostic skin biopsy of the trunk. Pulse methylprednisolone 1,000 mg intravenously for three consecutive days (Days 17–19 of Month 5) was administered, with only transient stabilization of the cutaneous disease.

Histopathology revealed moderately atrophic epidermis with interface dermatitis, basement membrane thickening, diffuse keratinocyte apoptosis, hyperkeratosis, and superficial perivascular lymphocytic infiltrates. Direct immunofluorescence showed focal IgG deposits along the basement membrane zone (positive lupus band test), providing histopathological confirmation of subacute cutaneous lupus erythematosus at Month 9. The differential diagnosis included progression of cryoglobulinemic vasculitis, drug eruption, viral exanthem, and photosensitivity reaction. Importantly, the possibility of SCLE occurring in the context of Sjögren’s disease (rather than as a drug-induced phenomenon) was considered; however, the absence of prior photosensitivity despite longstanding anti-Ro/SSA positivity, the temporal relationship between rituximab administration and cutaneous onset (Month 4), and the clinical course following B-cell depletion collectively supported a probable diagnosis of drug-induced SCLE. The causal link with rituximab, while strongly suggested by the temporal association and clinical context, is presented as probable rather than definitive, given the pre-existing Sjögren’s disease background and persistent anti-Ro/SSA seropositivity.

Given biopsy-confirmed SCLE and concomitant active cryoglobulinemic vasculitis refractory to B-cell depletion and high-dose corticosteroids, a treatment strategy targeting the type I interferon pathway was adopted at Month 9. Anifrolumab 300 mg was started as an intravenous infusion every 4 weeks, while hydroxychloroquine 400 mg/day was continued and methylprednisolone tapered to 6 mg/day. The first infusion was well tolerated, without infusion reactions or immediate adverse events.

Following anifrolumab initiation, the patient experienced rapid and marked clinical improvement. SCLE lesions on the face, trunk, and décolleté resolved completely. Vasculitic purpura improved substantially following anifrolumab, although mild residual activity persisted on the lower limbs, prompting the addition of mycophenolate mofetil 1,000 mg/day (500 mg twice daily) at Month 12, which resulted in complete remission of vasculitic manifestations. Complete disease control, with only post-inflammatory dyschromia, was thus achieved with the anifrolumab-based regimen. She has since remained in remission for 9 months on maintenance therapy with anifrolumab 300 mg intravenously every 4 weeks, mycophenolate mofetil 1,000 mg/day, hydroxychloroquine 400 mg/day, and minimal-dose corticosteroids, without serious infections, cytopenias, or other treatment-related complications and with full recovery of baseline functional status.

## Diagnostic assessment

### Histopathological examination and diagnosis

The biopsy specimen consisted of a skin fragment measuring 14 × 6 × 4 mm with whitish surface and areas of pigmentation. The specimen was bisected and processed in one tissue block at two levels for microscopic examination.

**FIGURE 3:**
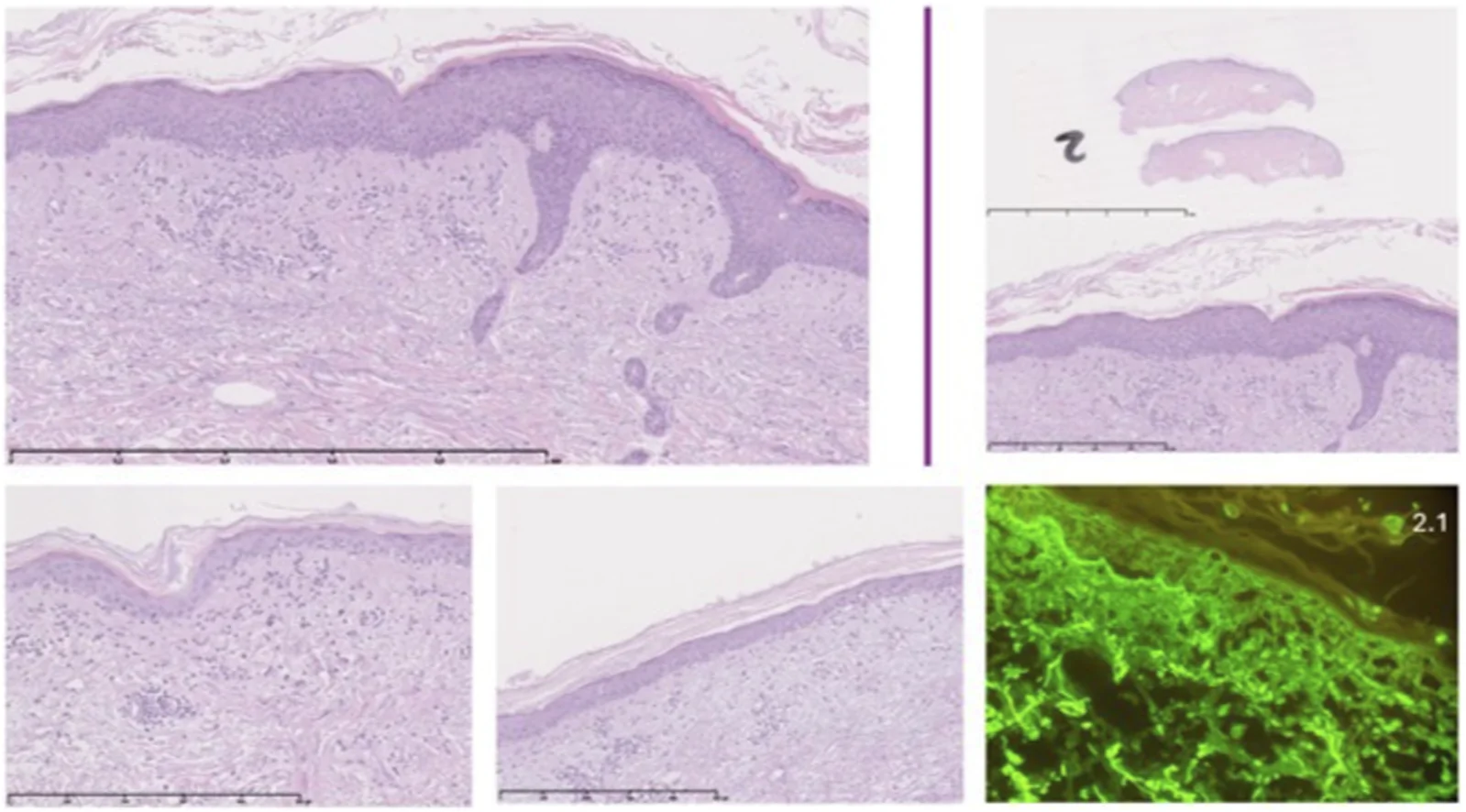
Histopathological features of rituximab-induced subacute cutaneous lupus erythematosus. Multiple representative microscopic fields (hematoxylin and eosin staining) at various magnifications demonstrating: moderately atrophic epidermis with interface dermatitis characterized by lymphocytic infiltrate at the dermal-epidermal junction; thickening of the basement membrane zone; focal vacuolar changes with basal keratinocyte degeneration; diffuse apoptotic keratinocytes; hyperkeratosis; and perivascular lymphocytic infiltration in the superficial dermis. The panoramic view (top right) shows the overall architecture of the biopsy specimen. Panel 2.1: Positive lupus band test in lesional skin. Direct immunofluorescence microscopy demonstrated continuous granular deposition of IgG along the dermal-epidermal junction (basement membrane zone). These histomorphological findings, in conjunction with positive direct immunofluorescence for IgG deposition along the basement membrane zone (lupus band test), are diagnostic of subacute cutaneous lupus erythematosus.

Histological examination revealed skin with intact but moderately atrophic epidermis (Figure 3). A discrete lymphocytic infiltrate was present at the dermal-epidermal junction. Characteristic findings included thickening of the basement membrane with focal aspects of interface dermatitis. Diffuse phenomena of keratinocyte apoptosis and hyperkeratosis were observed throughout the specimen. The superficial dermis demonstrated perivascular lymphocytic infiltration. Direct immunofluorescence microscopy demonstrated positive staining for focal IgG deposits along the basement membrane zone, consistent with a positive lupus band test (Figure 3, panel 2.1). The constellation of histomorphological findings was consistent with subacute cutaneous lupus erythematosus (SCLE).

### Differential diagnosis considerations

The differential diagnosis included progression of cryoglobulinemic vasculitis, drug eruption, viral exanthem, and photosensitivity reaction. A critical diagnostic consideration was the distinction between drug-induced SCLE and SCLE occurring as a manifestation of the underlying Sjögren’s disease, given the patient’s longstanding anti-Ro/SSA and anti-SSB/La positivity. The absence of prior photosensitivity or cutaneous lupus manifestations despite years of follow-up for Sjögren’s disease, the temporal relationship between rituximab administration and cutaneous onset, and the clinical course following B-cell depletion collectively supported a probable diagnosis of rituximab-induced DI-SCLE. Histopathological analysis of the purpuric lower extremity lesions was not performed in the context of this evaluation; the diagnosis of cryoglobulinemic vasculitis was based on clinical features and laboratory findings. Alternative diagnoses were considered unlikely based on biopsy findings and overall clinical course.

## Patient perspective

The patient reported that the erythematous rash on her face, chest, and back had substantially affected her daily social interactions and self-image prior to treatment. The psychological burden of visible facial involvement was described as particularly significant.

Following treatment with anifrolumab, she noted marked improvement in her skin condition within the first month, with progressive resolution over subsequent weeks. She remains satisfied with her maintenance therapy, reporting excellent disease control without significant impact on her daily activities or quality of life.

## Discussion

This case report describes a rare manifestation of rituximab-induced subacute cutaneous lupus erythematosus in a patient with primary Sjögren’s disease and illustrates the therapeutic utility of an anifrolumab-based regimen in achieving complete remission of both drug-induced cutaneous lupus and Sjögren’s disease-associated cryoglobulinemic vasculitis. The temporal relationship between rituximab administration and SCLE onset, combined with characteristic histopathological findings and pre-existing Ro/SS-A autoantibodies, supports a probable diagnosis of drug-induced SCLE; the causal link with rituximab is strongly suggested but should be considered probable rather than definitive, given the underlying Sjögren’s disease and persistent anti-Ro/SSA seropositivity.

Rituximab-associated hypersensitivity reactions encompass a spectrum ranging from immediate infusion-related reactions to delayed type IV hypersensitivity, including drug-induced lupus erythematosus (Isabwe et al., 2018; Galvão and Castells, 2015). Drug-induced SCLE is clinically, histopathologically, and immunologically indistinguishable from idiopathic SCLE, presenting with interface dermatitis and a positive lupus band test in approximately three-quarters of cases (Lowe et al., 2011). The incubation period varies considerably by drug class, with biologic agents typically demonstrating onset at several weeks after treatment (Lowe et al., 2011; Callen, 2010; Lis-Święty et al., 2013). Ro/SS-A autoantibodies are present in the majority of DI-SCLE cases and frequently persist after resolution (Lowe et al., 2011). The presence of underlying Sjögren’s disease, as in our patient, is a recognized predisposing factor, reflecting shared immunogenetic background (HLA-DR2/DR3) and Ro/SS-A autoantibody production (Lowe et al., 2011; Provost et al., 2007; Miyagawa et al., 1991; Röhrs et al., 2002).

The paradoxical induction of autoimmunity by rituximab is mechanistically attributed to compensatory BAFF elevation following B-cell depletion, which may promote survival and expansion of autoreactive clones (Stasi et al., 2008; Cambridge et al., 2006; Ramsköld et al., 2019; Mackay and Schneider, 2009); in our patient, SCLE onset at Month 4, coinciding with the nadir of B-cell depletion, is consistent with this hypothesis, though the absence of serial BAFF measurements limits its direct support. The underlying Sjögren’s disease background and persistent anti-Ro/SSA seropositivity likely further predisposed to this immunological cascade.

The selection of anifrolumab, a fully human monoclonal antibody targeting IFNAR1, represented a pathophysiologically rational approach to address both the rituximab-induced SCLE and the underlying Sjögren’s disease-associated vasculitis. While most DI-SCLE cases resolve spontaneously (Lowe et al., 2011), refractory cases may require conventional therapies including corticosteroids, hydroxychloroquine, or mycophenolate mofetil (Lowe et al., 2011; Cetkovská and Pizinger, 2006). Our patient’s incomplete response to pulse methylprednisolone, persistent SCLE manifestations, and concurrent active cryoglobulinemic vasculitis despite rituximab therapy highlighted the need for an alternative therapeutic strategy.

Anifrolumab has demonstrated efficacy in systemic lupus erythematosus, including significant improvement in cutaneous manifestations, through blockade of type I interferon signaling (Furie et al., 2019; Morand et al., 2020). In our patient, anifrolumab achieved complete remission of rituximab-induced SCLE. Complete remission of cryoglobulinemic vasculitis was subsequently obtained following the addition of mycophenolate mofetil, and the overall disease control should therefore be attributed to the anifrolumab-based combination regimen rather than to anifrolumab monotherapy. Our observations suggest that anifrolumab may be effective in managing SCLE regardless of etiology, extending previous evidence from idiopathic SCLE to drug-induced forms. The resolution of Sjögren’s disease-associated vasculitis within the context of this regimen supports the therapeutic potential of type I interferon blockade in systemic Sjögren’s disease, particularly for extraglandular manifestations refractory to B-cell depletion.

This case provides comprehensive documentation of a rare rituximab complication with detailed clinical photography, histopathological confirmation including direct immunofluorescence, and long-term follow-up demonstrating sustained remission. Limitations include the absence of serial BAFF measurements and B-cell subset analysis, which would have further supported the proposed BAFF-mediated mechanism. Additionally, serial measurement of type I interferon-regulated gene expression before and after anifrolumab therapy would have provided molecular confirmation of interferon pathway modulation. Histopathological analysis of the purpuric lower extremity lesions was not performed; the diagnosis of cryoglobulinemic vasculitis was based on clinical features and laboratory findings, and the absence of biopsy confirmation represents a further limitation. The combination therapy with mycophenolate mofetil, while clinically indicated for persistent vasculitic activity, limits attribution of therapeutic benefit for vasculitis exclusively to anifrolumab, though the temporal sequence and the complete resolution of SCLE preceding MMF addition suggest anifrolumab as the primary driver of cutaneous remission. The relatively short follow-up period limits assessment of long-term durability of response.

## Take-away lessons

Clinicians should maintain vigilance for paradoxical cutaneous reactions in patients receiving B-cell depleting therapies, particularly those with underlying Sjögren’s disease and anti-Ro/SSA positivity. New-onset photosensitive rashes in this context warrant prompt dermatological evaluation and biopsy, with careful differential diagnosis between drug-induced SCLE and cutaneous manifestations of the underlying connective tissue disease. Type I interferon blockade with anifrolumab represents a pathophysiologically rational option for refractory DI-SCLE and, within a combination regimen, for interferon-driven extraglandular Sjögren’s disease manifestations unresponsive to conventional therapies including B-cell depletion.

The BAFF-mediated mechanism underlying rituximab-induced autoimmunity has important therapeutic implications. Compensatory BAFF elevation following B-cell depletion creates conditions favorable for autoreactive expansion, suggesting that combination strategies targeting both B cells and downstream interferon signaling pathways may prove superior to B-cell depletion alone in preventing paradoxical autoimmune complications in susceptible individuals.

## Data Availability

The datasets presented in this article are not publicly available due to concerns regarding patient anonymity. Requests to access the datasets should be directed to the corresponding author.

## Nomenclature

BAFF: B-Cell Activating Factor
C3: Complement Component 3
C4: Complement Component 4
CRP: C-Reactive Protein
DILE: Drug-Induced Lupus Erythematosus
DI-SCLE: Drug-Induced Subacute Cutaneous Lupus Erythematosus
ESR: Erythrocyte Sedimentation Rate
HLA: Human Leukocyte Antigen
IFNAR1: Type I Interferon Receptor Subunit 1
IgG: Immunoglobulin G; IV, Intravenous
MMF: Mycophenolate Mofetil
PBMC: Peripheral Blood Mononuclear Cells
RF: Rheumatoid Factor
SCLE: Subacute Cutaneous Lupus Erythematosus.
